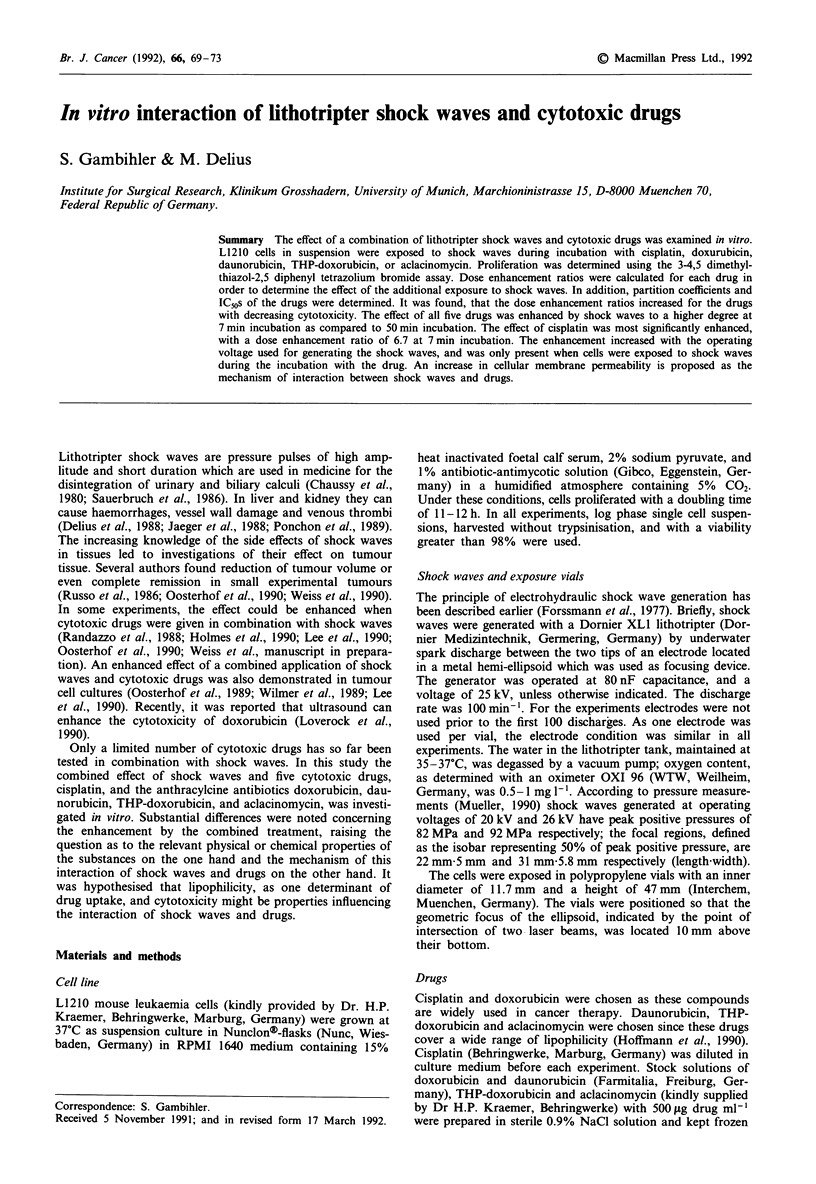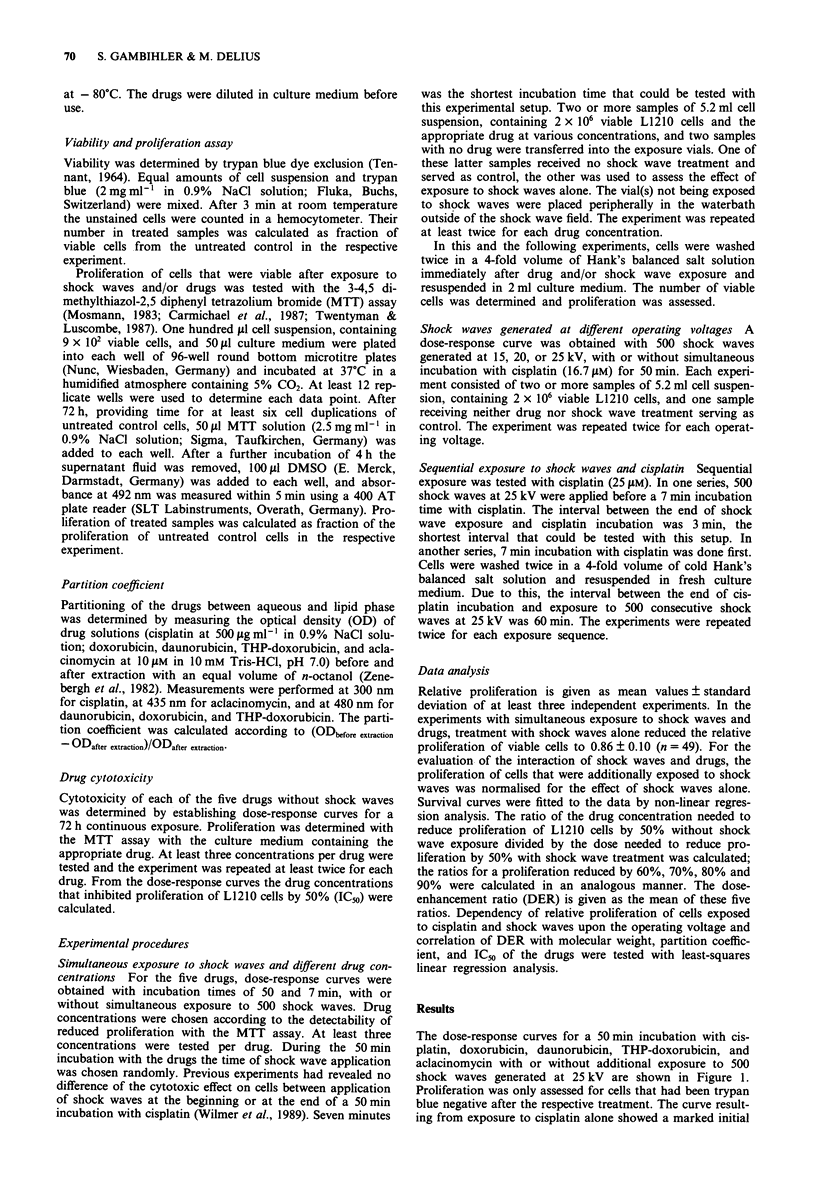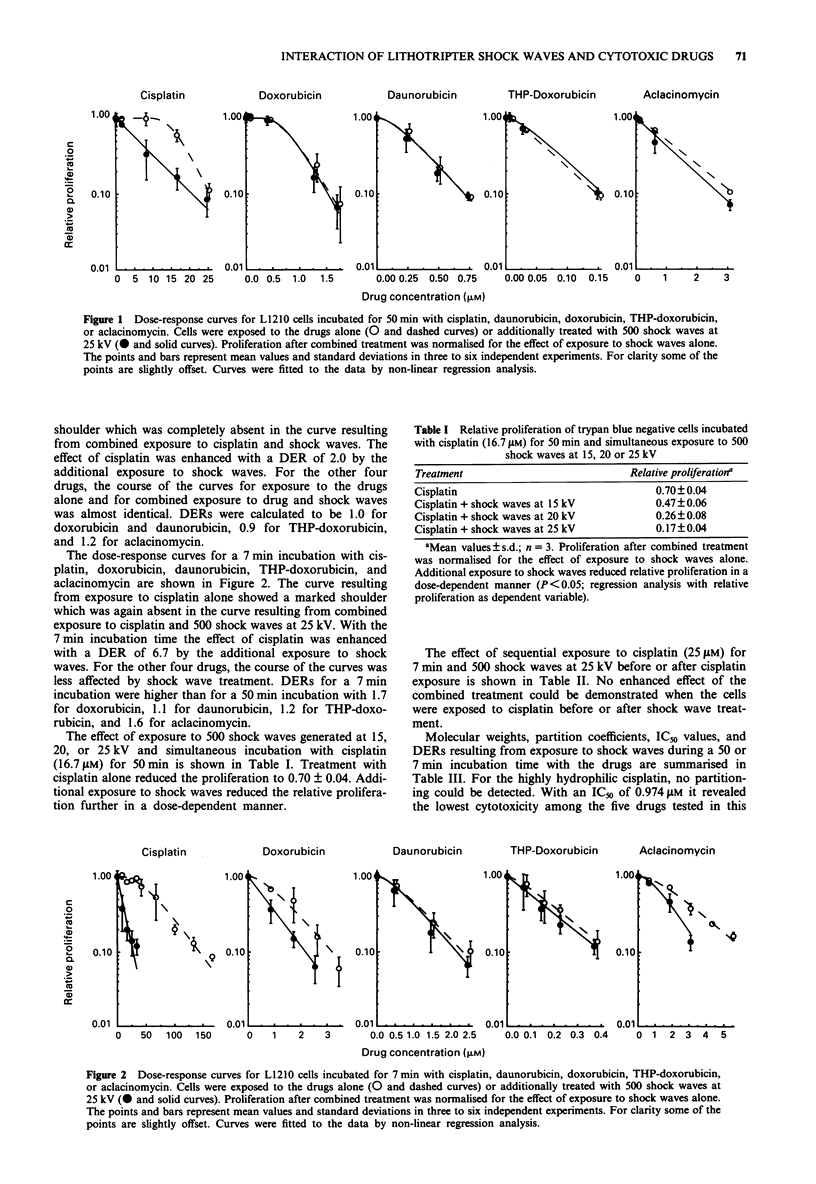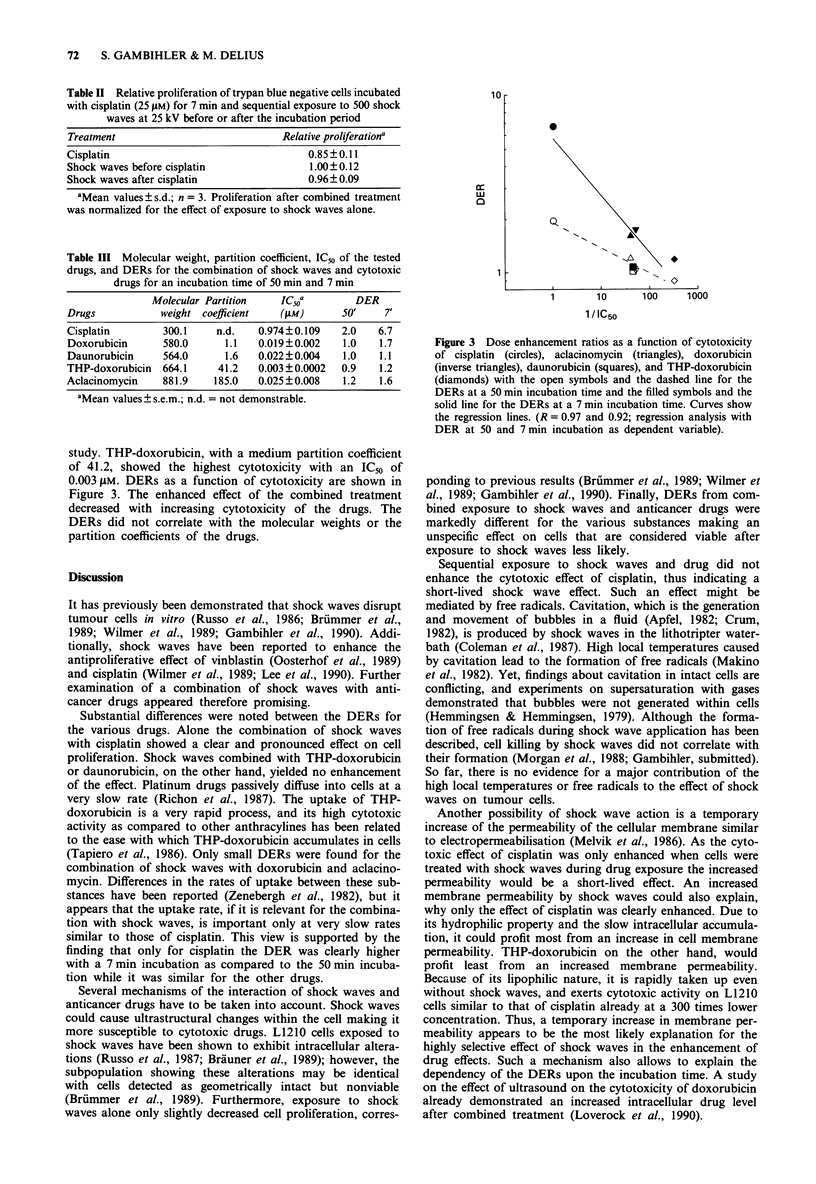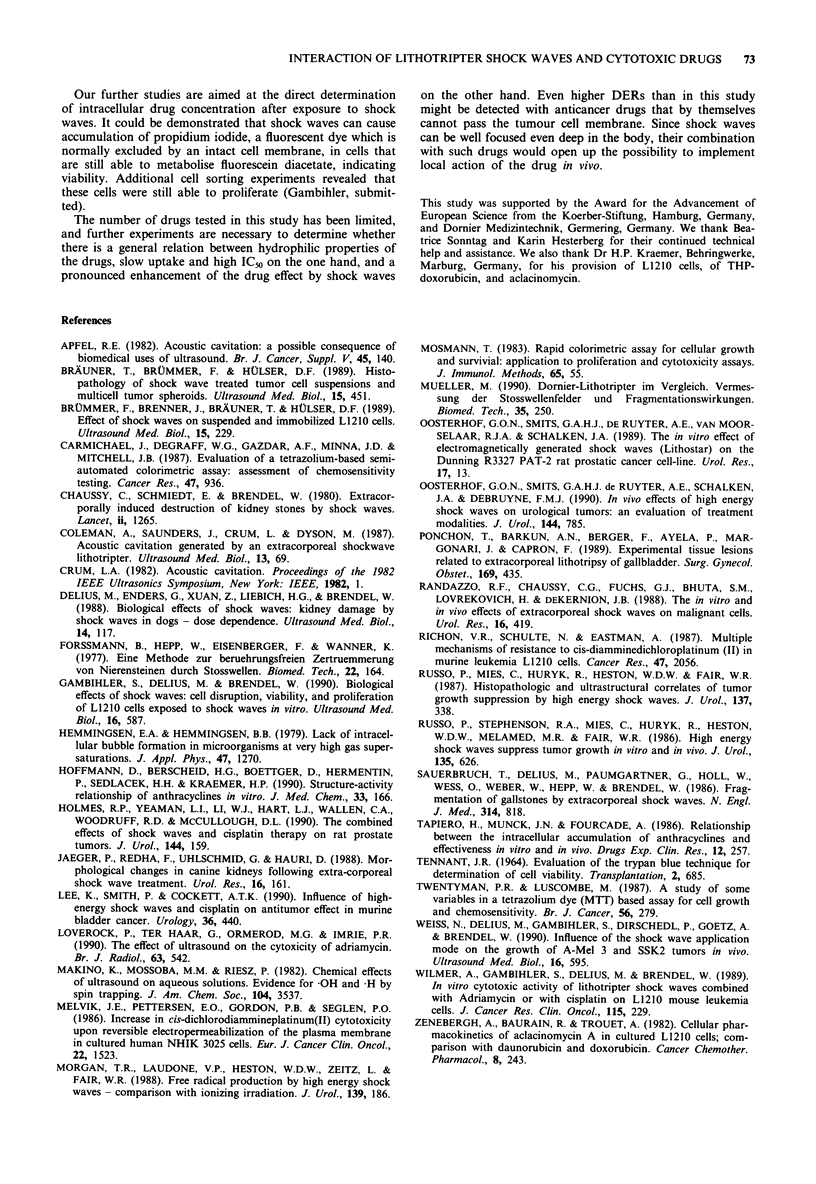# In vitro interaction of lithotripter shock waves and cytotoxic drugs.

**DOI:** 10.1038/bjc.1992.218

**Published:** 1992-07

**Authors:** S. Gambihler, M. Delius

**Affiliations:** Institute for Surgical Research, Klinikum Grosshadern, University of Munich, Federal Republic of Germany.

## Abstract

The effect of a combination of lithotripter shock waves and cytotoxic drugs was examined in vitro. L1210 cells in suspension were exposed to shock waves during incubation with cislatin, doxorubicin, daunorubicin, THP-doxorubicin, or aclacinomycin. Proliferation was determined using the 3-4,5 dimethylthiazol-2,5 diphenyl tetrazolium bromide assay. Dose enhancement ratios were calculated for each drug in order to determine the effect of the additional exposure to shock waves. In addition, partition coefficients and IC50s of the drugs were determined. It was found, that the dose enhancement ratios increased for the drugs with decreasing cytotoxicity. The effect of all five drugs was enhanced by shock waves to a higher degree at 7 min incubation as compared to 50 min incubation. The effect of cisplatin was most significantly enhanced, with a dose enhancement ratio of 6.7 at 7 min incubation. The enhancement increased with the operating voltage used for generating the shock waves, and was only present when cells were exposed to shock waves during the incubation with the drug. An increase in cellular membrane permeability is proposed as the mechanism of interaction between shock waves and drugs.


 © Macmillan Press Ltd., 1992

### *In vitro* interaction of lithotripter shock waves and cytotoxic drugs

S. Gambihler & M. Delius

*Institute for Surgical Research, Klinikum Grosshadern, University of Munich, Marchioninistrasse 15, D-8000 Muenchen 70, Federal Republic of Germany.*

**Summary** The effect of a combination of lithotripter shock waves and cytotoxic drugs was examined *in vitro*. L1210 cells in suspension were exposed to shock waves during incubation with cisplatin, doxurubicin, daunorubicin, THP-doxorubicin, or aclacinomycin. Proliferation was determined using the 3-4,5 dimethylthiazol-2,5 diphenyl tetrazolium bromide assay. Dose enhancement ratios were calculated for each drug in order to determine the effect of the additional exposure to shock waves. In addition, partition coefficients and $IC_{50}$s of the drugs were determined. It was found, that the dose enhancement ratios increased for the drugs with decreasing cytotoxicity. The effect of all five drugs was enhanced by shock waves to a higher degree at 7 min incubation as compared to 50 min incubation. The effect of cisplatin was most significantly enhanced, with a dose enhancement ratio of 6.7 at 7 min incubation. The enhancement increased with the operating voltage used for generating the shock waves, and was only present when cells were exposed to shock waves during the incubation with the drug. An increase in cellular membrane permeability is proposed as the mechanism of interaction between shock waves and drugs.

Lithotripter shock waves are pressure pulses of high amplitude and short duration which are used in medicine for the disintegration of urinary and biliary calculi (Chaussy *et al.*, 1980; Sauerbruch *et al.*, 1986). In liver and kidney they can cause haemorrhages, vessel wall damage and venous thrombi (Delius *et al.*, 1988; Jaeger *et al.*, 1988; Ponchon *et al.*, 1989). The increasing knowledge of the side effects of shock waves in tissues led to investigations of their effect on tumour tissue. Several authors found reduction of tumour volume or even complete remission in small experimental tumours (Russo *et al.*, 1986; Oosterhof *et al.*, 1990; Weiss *et al.*, 1990). In some experiments, the effect could be enhanced when cytotoxic drugs were given in combination with shock waves (Randazzo *et al.*, 1988; Holmes *et al.*, 1990; Lee *et al.*, 1990; Oosterhof *et al.*, 1990; Weiss *et al.*, manuscript in preparation). An enhanced effect of a combined application of shock waves and cytotoxic drugs was also demonstrated in tumour cell cultures (Oosterhof *et al.*, 1989; Wilmer *et al.*, 1989; Lee *et al.*, 1990). Recently, it was reported that ultrasound can enhance the cytotoxicity of doxorubicin (Loverock *et al.*, 1990).

Only a limited number of cytotoxic drugs has so far been tested in combination with shock waves. In this study the combined effect of shock waves and five cytotoxic drugs, cisplatin, and the anthracylcine antibiotics doxorubicin, daunorubicin, THP-doxorubicin, and aclacinomycin, was investigated *in vitro*. Substantial differences were noted concerning the enhancement by the combined treatment, raising the question as to the relevant physical or chemical properties of the substances on the one hand and the mechanism of this interaction of shock waves and drugs on the other hand. It was hypothesised that lipophilicity, as one determinant of drug uptake, and cytotoxicity might be properties influencing the interaction of shock waves and drugs.

### Materials and methods

### *Cell line*

L1210 mouse leukaemia cells (kindly provided by Dr. H.P. Kraemer, Behringwerke, Marburg, Germany) were grown at 37°C as suspension culture in Nunclon®-flasks (Nunc, Wiesbaden, Germany) in RPMI 1640 medium containing 15% heat inactivated foetal calf serum, 2% sodium pyruvate, and 1% antibiotic-antimycotic solution (Gibco, Eggenstein, Germany) in a humidified atmosphere containing 5% $CO_2$. Under these conditions, cells proliferated with a doubling time of 11-12 h. In all experiments, log phase single cell suspensions, harvested without trypsinisation, and with a viability greater than 98% were used.

### *Shock waves and exposure vials*

The principle of electrohydraulic shock wave generation has been described earlier (Forssmann *et al.*, 1977). Briefly, shock waves were generated with a Dornier XL1 lithotripter (Dornier Medizintechnik, Germering, Germany) by underwater spark discharge between the two tips of an electrode located in a metal hemi-ellipsoid which was used as focusing device. The generator was operated at 80 nF capacitance, and a voltage of 25 kV, unless otherwise indicated. The discharge rate was 100 $min^{-1}$. For the experiments electrodes were not used prior to the first 100 discharges. As one electrode was used per vial, the electrode condition was similar in all experiments. The water in the lithotripter tank, maintained at 35-37°C, was degassed by a vacuum pump; oxygen content, as determined with an oximeter OXI 96 (WTW, Weilheim, Germany, was 0.5-1 mg $l^{-1}$. According to pressure measurements (Mueller, 1990) shock waves generated at operating voltages of 20 kV and 26 kV have peak positive pressures of 82 MPa and 92 MPa respectively; the focal regions, defined as the isobar representing 50% of peak positive pressure, are 22 mm·5 mm and 31 mm·5.8 mm respectively (length·width).

The cells were exposed in polypropylene vials with an inner diameter of 11.7 mm and a height of 47 mm (Interchem, Muenchen, Germany). The vials were positioned so that the geometric focus of the ellipsoid, indicated by the point of intersection of two laser beams, was located 10 mm above their bottom.

### *Drugs*

Cisplatin and doxorubicin were chosen as these compounds are widely used in cancer therapy. Daunorubicin, THP-doxorubicin and aclacinomycin were chosen since these drugs cover a wide range of lipophilicity (Hoffmann *et al.*, 1990). Cisplatin (Behringwerke, Marburg, Germany) was diluted in culture medium before each experiment. Stock solutions of doxorubicin and daunorubicin (Farmitalia, Freiburg, Germany), THP-doxorubicin and aclacinomycin (kindly supplied by Dr H.P. Kraemer, Behringwerke) with 500 $\mu g$ drug $ml^{-1}$ were prepared in sterile 0.9% NaCl solution and kept frozen

Correspondence: S. Gambihler.
Received 5 November 1991; and in revised form 17 March 1992.

at − 80°C. The drugs were diluted in culture medium before use.

*Viability and proliferation assay*

Viability was determined by trypan blue dye exclusion (Tennant, 1964). Equal amounts of cell suspension and trypan blue ($2\ mg\ ml^{-1}$ in 0.9% NaCl solution; Fluka, Buchs, Switzerland) were mixed. After 3 min at room temperature the unstained cells were counted in a hemocytometer. Their number in treated samples was calculated as fraction of viable cells from the untreated control in the respective experiment.

Proliferation of cells that were viable after exposure to shock waves and/or drugs was tested with the 3-4,5 dimethylthiazol-2,5 diphenyl tetrazolium bromide (MTT) assay (Mosmann, 1983; Carmichael *et al.*, 1987; Twentyman & Luscombe, 1987). One hundred µl cell suspension, containing $9 \times 10^2$ viable cells, and 50 µl culture medium were plated into each well of 96-well round bottom microtitre plates (Nunc, Wiesbaden, Germany) and incubated at 37°C in a humidified atmosphere containing 5% $CO_2$. At least 12 replicate wells were used to determine each data point. After 72 h, providing time for at least six cell duplications of untreated control cells, 50 µl MTT solution ($2.5\ mg\ ml^{-1}$ in 0.9% NaCl solution; Sigma, Taufkirchen, Germany) was added to each well. After a further incubation of 4 h the supernatant fluid was removed, 100 µl DMSO (E. Merck, Darmstadt, Germany) was added to each well, and absorbance at 492 nm was measured within 5 min using a 400 AT plate reader (SLT Labinstruments, Overath, Germany). Proliferation of treated samples was calculated as fraction of the proliferation of untreated control cells in the respective experiment.

*Partition coefficient*

Partitioning of the drugs between aqueous and lipid phase was determined by measuring the optical density (OD) of drug solutions (cisplatin at $500\ \mu g\ ml^{-1}$ in 0.9% NaCl solution; doxorubicin, daunorubicin, THP-doxorubicin, and aclacinomycin at 10 µM in 10 mM Tris-HCl, pH 7.0) before and after extraction with an equal volume of *n*-octanol (Zenebergh *et al.*, 1982). Measurements were performed at 300 nm for cisplatin, at 435 nm for aclacinomycin, and at 480 nm for daunorubicin, doxorubicin, and THP-doxorubicin. The partition coefficient was calculated according to $(OD_{before\ extraction} - OD_{after\ extraction})/OD_{after\ extraction}$.

*Drug cytotoxicity*

Cytotoxicity of each of the five drugs without shock waves was determined by establishing dose-response curves for a 72 h continuous exposure. Proliferation was determined with the MTT assay with the culture medium containing the appropriate drug. At least three concentrations per drug were tested and the experiment was repeated at least twice for each drug. From the dose-response curves the drug concentrations that inhibited proliferation of L1210 cells by 50% ($IC_{50}$) were calculated.

*Experimental procedures*

*Simultaneous exposure to shock waves and different drug concentrations* For the five drugs, dose-response curves were obtained with incubation times of 50 and 7 min, with or without simultaneous exposure to 500 shock waves. Drug concentrations were chosen according to the detectability of reduced proliferation with the MTT assay. At least three concentrations were tested per drug. During the 50 min incubation with the drugs the time of shock wave application was chosen randomly. Previous experiments had revealed no difference of the cytotoxic effect on cells between application of shock waves at the beginning or at the end of a 50 min incubation with cisplatin (Wilmer *et al.*, 1989). Seven minutes was the shortest incubation time that could be tested with this experimental setup. Two or more samples of 5.2 ml cell suspension, containing $2 \times 10^6$ viable L1210 cells and the appropriate drug at various concentrations, and two samples with no drug were transferred into the exposure vials. One of these latter samples received no shock wave treatment and served as control, the other was used to assess the effect of exposure to shock waves alone. The vial(s) not being exposed to shock waves were placed peripherally in the waterbath outside of the shock wave field. The experiment was repeated at least twice for each drug concentration.

In this and the following experiments, cells were washed twice in a 4-fold volume of Hank's balanced salt solution immediately after drug and/or shock wave exposure and resuspended in 2 ml culture medium. The number of viable cells was determined and proliferation was assessed.

*Shock waves generated at different operating voltages* A dose-response curve was obtained with 500 shock waves generated at 15, 20, or 25 kV, with or without simultaneous incubation with cisplatin (16.7 µM) for 50 min. Each experiment consisted of two or more samples of 5.2 ml cell suspension, containing $2 \times 10^6$ viable L1210 cells, and one sample receiving neither drug nor shock wave treatment serving as control. The experiment was repeated twice for each operating voltage.

*Sequential exposure to shock waves and cisplatin* Sequential exposure was tested with cisplatin (25 µM). In one series, 500 shock waves at 25 kV were applied before a 7 min incubation time with cisplatin. The interval between the end of shock wave exposure and cisplatin incubation was 3 min, the shortest interval that could be tested with this setup. In another series, 7 min incubation with cisplatin was done first. Cells were washed twice in a 4-fold volume of cold Hank's balanced salt solution and resuspended in fresh culture medium. Due to this, the interval between the end of cisplatin incubation and exposure to 500 consecutive shock waves at 25 kV was 60 min. The experiments were repeated twice for each exposure sequence.

*Data analysis*

Relative proliferation is given as mean values ± standard deviation of at least three independent experiments. In the experiments with simultaneous exposure to shock waves and drugs, treatment with shock waves alone reduced the relative proliferation of viable cells to $0.86 \pm 0.10$ ($n = 49$). For the evaluation of the interaction of shock waves and drugs, the proliferation of cells that were additionally exposed to shock waves was normalised for the effect of shock waves alone. Survival curves were fitted to the data by non-linear regression analysis. The ratio of the drug concentration needed to reduce proliferation of L1210 cells by 50% without shock wave exposure divided by the dose needed to reduce proliferation by 50% with shock wave treatment was calculated; the ratios for a proliferation reduced by 60%, 70%, 80% and 90% were calculated in an analogous manner. The dose-enhancement ratio (DER) is given as the mean of these five ratios. Dependency of relative proliferation of cells exposed to cisplatin and shock waves upon the operating voltage and correlation of DER with molecular weight, partition coefficient, and $IC_{50}$ of the drugs were tested with least-squares linear regression analysis.

### Results

The dose-response curves for a 50 min incubation with cisplatin, doxorubicin, daunorubicin, THP-doxorubicin, and aclacinomycin with or without additional exposure to 500 shock waves generated at 25 kV are shown in Figure 1. Proliferation was only assessed for cells that had been trypan blue negative after the respective treatment. The curve resulting from exposure to cisplatin alone showed a marked initial

**Figure 1** Dose-response curves for L1210 cells incubated for 50 min with cisplatin, daunorubicin, doxorubicin, THP-doxorubicin, or aclacinomycin. Cells were exposed to the drugs alone (○ and dashed curves) or additionally treated with 500 shock waves at 25 kV (● and solid curves). Proliferation after combined treatment was normalised for the effect of exposure to shock waves alone. The points and bars represent mean values and standard deviations in three to six independent experiments. For clarity some of the points are slightly offset. Curves were fitted to the data by non-linear regression analysis.

shoulder which was completely absent in the curve resulting from combined exposure to cisplatin and shock waves. The effect of cisplatin was enhanced with a DER of 2.0 by the additional exposure to shock waves. For the other four drugs, the course of the curves for exposure to the drugs alone and for combined exposure to drug and shock waves was almost identical. DERs were calculated to be 1.0 for doxorubicin and daunorubicin, 0.9 for THP-doxorubicin, and 1.2 for aclacinomycin.

The dose-response curves for a 7 min incubation with cisplatin, doxorubicin, daunorubicin, THP-doxorubicin, and aclacinomycin are shown in Figure 2. The curve resulting from exposure to cisplatin alone showed a marked shoulder which was again absent in the curve resulting from combined exposure to cisplatin and 500 shock waves at 25 kV. With the 7 min incubation time the effect of cisplatin was enhanced with a DER of 6.7 by the additional exposure to shock waves. For the other four drugs, the course of the curves was less affected by shock wave treatment. DERs for a 7 min incubation were higher than for a 50 min incubation with 1.7 for doxorubicin, 1.1 for daunorubicin, 1.2 for THP-doxorubicin, and 1.6 for aclacinomycin.

The effect of exposure to 500 shock waves generated at 15, 20, or 25 kV and simultaneous incubation with cisplatin (16.7 μM) for 50 min is shown in Table I. Treatment with cisplatin alone reduced the proliferation to 0.70 ± 0.04. Additional exposure to shock waves reduced the relative proliferation further in a dose-dependent manner.

**Table I** Relative proliferation of trypan blue negative cells incubated with cisplatin (16.7 μM) for 50 min and simultaneous exposure to 500 shock waves at 15, 20 or 25 kV

| *Treatment* | *Relative proliferation*[a] |
|---|---|
| Cisplatin | 0.70 ± 0.04 |
| Cisplatin + shock waves at 15 kV | 0.47 ± 0.06 |
| Cisplatin + shock waves at 20 kV | 0.26 ± 0.08 |
| Cisplatin + shock waves at 25 kV | 0.17 ± 0.04 |

[a]Mean values ± s.d.; $n = 3$. Proliferation after combined treatment was normalised for the effect of exposure to shock waves alone. Additional exposure to shock waves reduced relative proliferation in a dose-dependent manner ($P < 0.05$; regression analysis with relative proliferation as dependent variable).

The effect of sequential exposure to cisplatin (25 μM) for 7 min and 500 shock waves at 25 kV before or after cisplatin exposure is shown in Table II. No enhanced effect of the combined treatment could be demonstrated when the cells were exposed to cisplatin before or after shock wave treatment.

Molecular weights, partition coefficients, $IC_{50}$ values, and DERs resulting from exposure to shock waves during a 50 or 7 min incubation time with the drugs are summarised in Table III. For the highly hydrophilic cisplatin, no partitioning could be detected. With an $IC_{50}$ of 0.974 μM it revealed the lowest cytotoxicity among the five drugs tested in this

**Figure 2** Dose-response curves for L1210 cells incubated for 7 min with cisplatin, daunorubicin, doxorubicin, THP-doxorubicin, or aclacinomycin. Cells were exposed to the drugs alone (○ and dashed curves) or additionally treated with 500 shock waves at 25 kV (● and solid curves). Proliferation after combined treatment was normalised for the effect of exposure to shock waves alone. The points and bars represent mean values and standard deviations in three to six independent experiments. For clarity some of the points are slightly offset. Curves were fitted to the data by non-linear regression analysis.

**Table II** Relative proliferation of trypan blue negative cells incubated with cisplatin (25 μM) for 7 min and sequential exposure to 500 shock waves at 25 kV before or after the incubation period

| *Treatment* | *Relative proliferation*[a] |
|---|---|
| Cisplatin | 0.85±0.11 |
| Shock waves before cisplatin | 1.00±0.12 |
| Shock waves after cisplatin | 0.96±0.09 |

[a]Mean values±s.d.; $n = 3$. Proliferation after combined treatment was normalized for the effect of exposure to shock waves alone.

**Table III** Molecular weight, partition coefficient, $IC_{50}$ of the tested drugs, and DERs for the combination of shock waves and cytotoxic drugs for an incubation time of 50 min and 7 min

| *Drugs* | *Molecular weight* | *Partition coefficient* | *IC*$_{50}$[a] *(μM)* | *DER 50′* | *DER 7′* |
|---|---|---|---|---|---|
| Cisplatin | 300.1 | n.d. | 0.974±0.109 | 2.0 | 6.7 |
| Doxorubicin | 580.0 | 1.1 | 0.019±0.002 | 1.0 | 1.7 |
| Daunorubicin | 564.0 | 1.6 | 0.022±0.004 | 1.0 | 1.1 |
| THP-doxorubicin | 664.1 | 41.2 | 0.003±0.0002 | 0.9 | 1.2 |
| Aclacinomycin | 881.9 | 185.0 | 0.025±0.008 | 1.2 | 1.6 |

[a]Mean values±s.e.m.; n.d. = not demonstrable.

**Figure 3** Dose enhancement ratios as a function of cytotoxicity of cisplatin (circles), aclacinomycin (triangles), doxorubicin (inverse triangles), daunorubicin (squares), and THP-doxorubicin (diamonds) with the open symbols and the dashed line for the DERs at a 50 min incubation time and the filled symbols and the solid line for the DERs at a 7 min incubation time. Curves show the regression lines. ($R = 0.97$ and 0.92; regression analysis with DER at 50 and 7 min incubation as dependent variable).

study. THP-doxorubicin, with a medium partition coefficient of 41.2, showed the highest cytotoxicity with an $IC_{50}$ of 0.003 μM. DERs as a function of cytotoxicity are shown in Figure 3. The enhanced effect of the combined treatment decreased with increasing cytotoxicity of the drugs. The DERs did not correlate with the molecular weights or the partition coefficients of the drugs.

### Discussion

It has previously been demonstrated that shock waves disrupt tumour cells *in vitro* (Russo *et al.*, 1986; Brümmer *et al.*, 1989; Wilmer *et al.*, 1989; Gambihler *et al.*, 1990). Additionally, shock waves have been reported to enhance the antiproliferative effect of vinblastin (Oosterhof *et al.*, 1989) and cisplatin (Wilmer *et al.*, 1989; Lee *et al.*, 1990). Further examination of a combination of shock waves with anticancer drugs appeared therefore promising.

Substantial differences were noted between the DERs for the various drugs. Alone the combination of shock waves with cisplatin showed a clear and pronounced effect on cell proliferation. Shock waves combined with THP-doxorubicin or daunorubicin, on the other hand, yielded no enhancement of the effect. Platinum drugs passively diffuse into cells at a very slow rate (Richon *et al.*, 1987). The uptake of THP-doxorubicin is a very rapid process, and its high cytotoxic activity as compared to other anthracylines has been related to the ease with which THP-doxorubicin accumulates in cells (Tapiero *et al.*, 1986). Only small DERs were found for the combination of shock waves with doxorubicin and aclacinomycin. Differences in the rates of uptake between these substances have been reported (Zenebergh *et al.*, 1982), but it appears that the uptake rate, if it is relevant for the combination with shock waves, is important only at very slow rates similar to those of cisplatin. This view is supported by the finding that only for cisplatin the DER was clearly higher with a 7 min incubation as compared to the 50 min incubation while it was similar for the other drugs.

Several mechanisms of the interaction of shock waves and anticancer drugs have to be taken into account. Shock waves could cause ultrastructural changes within the cell making it more susceptible to cytotoxic drugs. L1210 cells exposed to shock waves have been shown to exhibit intracellular alterations (Russo *et al.*, 1987; Bräuner *et al.*, 1989); however, the subpopulation showing these alterations may be identical with cells detected as geometrically intact but nonviable (Brümmer *et al.*, 1989). Furthermore, exposure to shock waves alone only slightly decreased cell proliferation, corresponding to previous results (Brümmer *et al.*, 1989; Wilmer *et al.*, 1989; Gambihler *et al.*, 1990). Finally, DERs from combined exposure to shock waves and anticancer drugs were markedly different for the various substances making an unspecific effect on cells that are considered viable after exposure to shock waves less likely.

Sequential exposure to shock waves and drug did not enhance the cytotoxic effect of cisplatin, thus indicating a short-lived shock wave effect. Such an effect might be mediated by free radicals. Cavitation, which is the generation and movement of bubbles in a fluid (Apfel, 1982; Crum, 1982), is produced by shock waves in the lithotripter waterbath (Coleman *et al.*, 1987). High local temperatures caused by cavitation lead to the formation of free radicals (Makino *et al.*, 1982). Yet, findings about cavitation in intact cells are conflicting, and experiments on supersaturation with gases demonstrated that bubbles were not generated within cells (Hemmingsen & Hemmingsen, 1979). Although the formation of free radicals during shock wave application has been described, cell killing by shock waves did not correlate with their formation (Morgan *et al.*, 1988; Gambihler, submitted). So far, there is no evidence for a major contribution of the high local temperatures or free radicals to the effect of shock waves on tumour cells.

Another possibility of shock wave action is a temporary increase of the permeability of the cellular membrane similar to electropermeabilisation (Melvik *et al.*, 1986). As the cytotoxic effect of cisplatin was only enhanced when cells were treated with shock waves during drug exposure the increased permeability would be a short-lived effect. An increased membrane permeability by shock waves could also explain, why only the effect of cisplatin was clearly enhanced. Due to its hydrophilic property and the slow intracellular accumulation, it could profit most from an increase in cell membrane permeability. THP-doxorubicin on the other hand, would profit least from an increased membrane permeability. Because of its lipophilic nature, it is rapidly taken up even without shock waves, and exerts cytotoxic activity on L1210 cells similar to that of cisplatin already at a 300 times lower concentration. Thus, a temporary increase in membrane permeability appears to be the most likely explanation for the highly selective effect of shock waves in the enhancement of drug effects. Such a mechanism also allows to explain the dependency of the DERs upon the incubation time. A study on the effect of ultrasound on the cytotoxicity of doxorubicin already demonstrated an increased intracellular drug level after combined treatment (Loverock *et al.*, 1990).

Our further studies are aimed at the direct determination of intracellular drug concentration after exposure to shock waves. It could be demonstrated that shock waves can cause accumulation of propidium iodide, a fluorescent dye which is normally excluded by an intact cell membrane, in cells that are still able to metabolise fluorescein diacetate, indicating viability. Additional cell sorting experiments revealed that these cells were still able to proliferate (Gambihler, submitted).

The number of drugs tested in this study has been limited, and further experiments are necessary to determine whether there is a general relation between hydrophilic properties of the drugs, slow uptake and high $IC_{50}$ on the one hand, and a pronounced enhancement of the drug effect by shock waves on the other hand. Even higher DERs than in this study might be detected with anticancer drugs that by themselves cannot pass the tumour cell membrane. Since shock waves can be well focused even deep in the body, their combination with such drugs would open up the possibility to implement local action of the drug *in vivo*.

This study was supported by the Award for the Advancement of European Science from the Koerber-Stiftung, Hamburg, Germany, and Dornier Medizintechnik, Germering, Germany. We thank Beatrice Sonntag and Karin Hesterberg for their continued technical help and assistance. We also thank Dr H.P. Kraemer, Behringwerke, Marburg, Germany, for his provision of L1210 cells, of THP-doxorubicin, and aclacinomycin.

### References

APFEL, R.E. (1982). Acoustic cavitation: a possible consequence of biomedical uses of ultrasound. *Br. J. Cancer, Suppl. V*, **45**, 140.

BRÄUNER, T., BRÜMMER, F. & HÜLSER, D.F. (1989). Histopathology of shock wave treated tumor cell suspensions and multicell tumor spheroids. *Ultrasound Med. Biol.*, **15**, 451.

BRÜMMER, F., BRENNER, J., BRÄUNER, T. & HÜLSER, D.F. (1989). Effect of shock waves on suspended and immobilized L1210 cells. *Ultrasound Med. Biol.*, **15**, 229.

CARMICHAEL, J., DEGRAFF, W.G., GAZDAR, A.F., MINNA, J.D. & MITCHELL, J.B. (1987). Evaluation of a tetrazolium-based semiautomated colorimetric assay: assessment of chemosensitivity testing. *Cancer Res.*, **47**, 936.

CHAUSSY, C., SCHMIEDT, E. & BRENDEL, W. (1980). Extracorporally induced destruction of kidney stones by shock waves. *Lancet*, **ii**, 1265.

COLEMAN, A., SAUNDERS, J., CRUM, L. & DYSON, M. (1987). Acoustic cavitation generated by an extracorporeal shockwave lithotripter. *Ultrasound Med. Biol.*, **13**, 69.

CRUM, L.A. (1982). Acoustic cavitation. *Proceedings of the 1982 IEEE Ultrasonics Symposium, New York: IEEE*, **1982**, 1.

DELIUS, M., ENDERS, G., XUAN, Z., LIEBICH, H.G., & BRENDEL, W. (1988). Biological effects of shock waves: kidney damage by shock waves in dogs – dose dependence. *Ultrasound Med. Biol.*, **14**, 117.

FORSSMANN, B., HEPP, W., EISENBERGER, F. & WANNER, K. (1977). Eine Methode zur beruehrungsfreien Zertruemmerung von Nierensteinen durch Stosswellen. *Biomed. Tech.*, **22**, 164.

GAMBIHLER, S., DELIUS, M. & BRENDEL, W. (1990). Biological effects of shock waves: cell disruption, viability, and proliferation of L1210 cells exposed to shock waves *in vitro*. *Ultrasound Med. Biol.*, **16**, 587.

HEMMINGSEN, E.A. & HEMMINGSEN, B.B. (1979). Lack of intracellular bubble formation in microorganisms at very high gas supersaturations. *J. Appl. Phys.*, **47**, 1270.

HOFFMANN, D., BERSCHEID, H.G., BOETTGER, D., HERMENTIN, P., SEDLACEK, H.H. & KRAEMER, H.P. (1990). Structure-activity relationship of anthracyclines *in vitro*. *J. Med. Chem.*, **33**, 166.

HOLMES, R.P., YEAMAN, L.I., LI, W.J., HART, L.J., WALLEN, C.A., WOODRUFF, R.D. & MCCULLOUGH, D.L. (1990). The combined effects of shock waves and cisplatin therapy on rat prostate tumors. *J. Urol.*, **144**, 159.

JAEGER, P., REDHA, F., UHLSCHMID, G. & HAURI, D. (1988). Morphological changes in canine kidneys following extra-corporeal shock wave treatment. *Urol. Res.*, **16**, 161.

LEE, K., SMITH, P. & COCKETT, A.T.K. (1990). Influence of high-energy shock waves and cisplatin on antitumor effect in murine bladder cancer. *Urology*, **36**, 440.

LOVEROCK, P., TER HAAR, G., ORMEROD, M.G. & IMRIE, P.R. (1990). The effect of ultrasound on the cytoxicity of adriamycin. *Br. J. Radiol.*, **63**, 542.

MAKINO, K., MOSSOBA, M.M. & RIESZ, P. (1982). Chemical effects of ultrasound on aqueous solutions. Evidence for ·OH and ·H by spin trapping. *J. Am. Chem. Soc.*, **104**, 3537.

MELVIK, J.E., PETTERSEN, E.O., GORDON, P.B. & SEGLEN, P.O. (1986). Increase in *cis*-dichlorodiammineplatinum(II) cytotoxicity upon reversible electropermeabilization of the plasma membrane in cultured human NHIK 3025 cells. *Eur. J. Cancer Clin. Oncol.*, **22**, 1523.

MORGAN, T.R., LAUDONE, V.P., HESTON, W.D.W., ZEITZ, L. & FAIR, W.R. (1988). Free radical production by high energy shock waves – comparison with ionizing irradiation. *J. Urol.*, **139**, 186.

MOSMANN, T. (1983). Rapid colorimetric assay for cellular growth and survivial: application to proliferation and cytotoxicity assays. *J. Immunol. Methods*, **65**, 55.

MUELLER, M. (1990). Dornier-Lithotripter im Vergleich. Vermessung der Stosswellenfelder und Fragmentationswirkungen. *Biomed. Tech.*, **35**, 250.

OOSTERHOF, G.O.N., SMITS, G.A.H.J., DE RUYTER, A.E., VAN MOORSELAAR, R.J.A. & SCHALKEN, J.A. (1989). The *in vitro* effect of electromagnetically generated shock waves (Lithostar) on the Dunning R3327 PAT-2 rat prostatic cancer cell-line. *Urol. Res.*, **17**, 13.

OOSTERHOF, G.O.N., SMITS, G.A.H.J. de RUYTER, A.E., SCHALKEN, J.A. & DEBRUYNE, F.M.J. (1990). *In vivo* effects of high energy shock waves on urological tumors: an evaluation of treatment modalities. *J. Urol.*, **144**, 785.

PONCHON, T., BARKUN, A.N., BERGER, F., AYELA, P., MARGONARI, J. & CAPRON, F. (1989). Experimental tissue lesions related to extracorporeal lithotripsy of gallbladder. *Surg. Gynecol. Obstet.*, **169**, 435.

RANDAZZO, R.F., CHAUSSY, C.G., FUCHS, G.J., BHUTA, S.M., LOVREKOVICH, H. & DEKERNION, J.B. (1988). The *in vitro* and *in vivo* effects of extracorporeal shock waves on malignant cells. *Urol. Res.*, **16**, 419.

RICHON, V.R., SCHULTE, N. & EASTMAN, A. (1987). Multiple mechanisms of resistance to cis-diamminedichloroplatinum (II) in murine leukemia L1210 cells. *Cancer Res.*, **47**, 2056.

RUSSO, P., MIES, C., HURYK, R., HESTON, W.D.W. & FAIR, W.R. (1987). Histopathologic and ultrastructural correlates of tumor growth suppression by high energy shock waves. *J. Urol.*, **137**, 338.

RUSSO, P., STEPHENSON, R.A., MIES, C., HURYK, R., HESTON, W.D.W., MELAMED, M.R. & FAIR, W.R. (1986). High energy shock waves suppress tumor growth *in vitro* and *in vivo*. *J. Urol.*, **135**, 626.

SAUERBRUCH, T., DELIUS, M., PAUMGARTNER, G., HOLL, W., WESS, O., WEBER, W., HEPP, W. & BRENDEL, W. (1986). Fragmentation of gallstones by extracorporeal shock waves. *N. Engl. J. Med.*, **314**, 818.

TAPIERO, H., MUNCK, J.N. & FOURCADE, A. (1986). Relationship between the intracellular accumulation of anthracyclines and effectiveness *in vitro* and *in vivo*. *Drugs Exp. Clin. Res.*, **12**, 257.

TENNANT, J.R. (1964). Evaluation of the trypan blue technique for determination of cell viability. *Transplantation*, **2**, 685.

TWENTYMAN, P.R. & LUSCOMBE, M. (1987). A study of some variables in a tetrazolium dye (MTT) based assay for cell growth and chemosensitivity. *Br. J. Cancer*, **56**, 279.

WEISS, N., DELIUS, M., GAMBIHLER, S., DIRSCHEDL, P., GOETZ, A. & BRENDEL, W. (1990). Influence of the shock wave application mode on the growth of A-Mel 3 and SSK2 tumors *in vivo*. *Ultrasound Med. Biol.*, **16**, 595.

WILMER, A., GAMBIHLER, S., DELIUS, M. & BRENDEL, W. (1989). *In vitro* cytotoxic activity of lithotripter shock waves combined with Adriamycin or with cisplatin on L1210 mouse leukemia cells. *J. Cancer Res. Clin. Oncol.*, **115**, 229.

ZENEBERGH, A., BAURAIN, R. & TROUET, A. (1982). Cellular pharmacokinetics of aclacinomycin A in cultured L1210 cells; comparison with daunorubicin and doxorubicin. *Cancer Chemother. Pharmacol.*, **8**, 243.